# Active Colitis-Induced Atrial Electrophysiological Remodeling

**DOI:** 10.3390/biom15070982

**Published:** 2025-07-10

**Authors:** Hiroki Kittaka, Edward J. Ouille V, Carlos H. Pereira, Andrès F. Pélaez, Ali Keshavarzian, Kathrin Banach

**Affiliations:** 1 Department of Internal Medicine/Cardiology, Rush University Medical Center, 1750 W. Harrison St., Chicago, IL 60612, USA; hiroki_kittaka@rush.edu (H.K.); eddieouille5@gmail.com (E.J.O.V.); h85.carlos@gmail.com (C.H.P.); apelaez@uwalumni.com (A.F.P.); 2 Rush Center for Integrated Microbiome and Chronobiology Research, Rush University Medical Center, 1725 W. Harrison St., Chicago, IL 60612, USA; ali_keshavarzian@rush.edu; 3 Department of Internal Medicine/Gastroenterology, Rush University Medical Center, 1725 W. Harrison St., Chicago, IL 60612, USA

**Keywords:** colitis, atrial myocyte, action potential, Na_v_1.5, K_v_4.2, Angiotensin II, atrial arrhythmia

## Abstract

Patients with ulcerative colitis exhibit an increased risk for supraventricular arrhythmia during the active disease phase of the disease and show signs of atrial electrophysiological remodeling in remission. The goal of this study was to determine the basis for colitis-induced changes in atrial excitability. In a mouse model (C57BL/6; 3 months) of dextran sulfate sodium (DSS)-induced active colitis (3.5% weight/volume, 7 days), electrocardiograms (ECG) revealed altered atrial electrophysiological properties with a prolonged P-wave duration and PR interval. ECG changes coincided with a decreased atrial conduction velocity in Langendorff perfused hearts. Action potentials (AP) recorded from isolated atrial myocytes displayed an attenuated maximal upstroke velocity and amplitude during active colitis, as well as a prolonged AP duration (APD). Voltage clamp analysis revealed a colitis-induced shift in the voltage-dependent activation of the Na-current (I_Na_) to more depolarizing voltages. In addition, protein levels of Na_v_1.5 protein and connexin isoform Cx43 were reduced. APD prolongation depended on a reduction in the transient outward K-current (I_to_) mostly generated by K_v_4.2 channels. The changes in ECG, atrial conductance, and APD were reversible upon remission. The change in conduction velocity predominantly depended on the reversibility of the reduced Cx43 and Na_v_1.5 expression. Treatment of mice with inhibitors of Angiotensin-converting enzyme (ACE) or Angiotensin II (AngII) receptor type 1 (AT1R) prevented the colitis-induced atrial electrophysiological remodeling. Our data support a colitis-induced increase in AngII signaling that promotes atrial electrophysiological remodeling and puts colitis patients at an increased risk for atrial arrhythmia.

## 1. Introduction

Ulcerative colitis (UC), one of the most common types of inflammatory bowel disease (IBD), is a chronic inflammation of the mucosa of the large intestine (colon) that is characterized by a relapsing and remitting course [1,2]. Colonic inflammation results in mucosa ulceration, colonic barrier disruption, bacterial translocation, and systemic inflammatory/immune reactions [1,2]. Indeed, IBD is an immune-mediated disease that affects organs distant from the gastrointestinal tract [3]. In the cardiovascular system, it manifests with inflammatory processes (e.g., pericarditis), arrhythmia, conduction disorders, and an increased risk for stroke [4,5,6,7]. Patients with IBD exhibit a 1.5 to 2-times higher risk for atrial fibrillation (AF) [7,8,9,10] that occurs at a younger age and in the absence of traditional cardiovascular risk factors. Episodes of AF occur during UC flares [8,9], but even in remission, patients exhibit changes in atrial electromechanical properties reflected in increased P-wave duration (P_dur_), P-wave dispersion (P_disp_), atrial electromechanical delay, and reduced left atrial mechanical function [6,11,12,13,14]. The ECG parameter P_dur_, P_disp_, as well as the P wave amplitude are independent predictors of an increased risk for AF [15,16,17]. The mechanisms of the colitis-induced atrial electrophysiological remodeling, or the electrophysiological phenotype underlying the ECG changes, have yet to be determined.

Alterations in P_dur_ and P_disp_ reflect changes in the intra- and inter- atrial conductance and a heterogeneity of the atrial excitation [17,18]. Atrial conduction velocity and dispersion depend on the upstroke velocity of the action potential (AP) driven by the activation of the voltage-gated sodium (Na) channels Na_v_1.5 (*SCN5A*), the intercellular coupling through the predominant atrial gap junction proteins connexin (Cx) 43 and Cx40, the AP duration (APD), and the degree of atrial fibrotic remodeling [19,20]. A reduced Na-current (I_Na_) density as well as a shift of the channels voltage dependence of activation impair the atrial conduction and lead to a prolonged and/or more heterogeneous cardiac excitation [21,22]. Altered Na_v_1.5 expression as well as gain and loss of function mutations in *SCN5A* have been linked to an increased propensity for AF [23,24,25]. The APD, on the other hand, depends on the balance between the voltage-dependent activation and inactivation of depolarizing currents such as the late I_Na_ (I_Na,L_) and the current through the L-type Calcium channel (LTCC), as well as the activation of re-polarizing K-channels. At this point, it is not known how changes in atrial ion channel expression or function contribute to the observed electrophysiological changes observed in patients with active colitis.

During active colitis, patients and animal models exhibit gut dysbiosis, bacterial translocation, increased intestinal and systemic inflammation, and increased sympathetic signaling [1,2]. These changes can promote the remodeling of atrial electrophysiological properties through increased levels of inflammatory cytokines such as interleukin-1 β (IL-1β), IL-17, IL-6, tumor necrosis factor-alpha (TNF-α), and interferon-gamma (IFN-γ), changes in gut-derived metabolites such as short chain fatty acids (SCFA) and trimethylamine N-oxide (TMAO), or the elevated transfer of lipopolysaccharide (LPS) into systemic circulation [26]. Interestingly, all components also alter the renin-angiotensin system (RAS), a key mediator of inflammatory signaling [27]. An increase in angiotensinogen (Agt) expression during colitis could therefore occur through increased sympathetic signaling [28], inflammatory cytokines [29], increased renin secretion through reduced SCFAs, and enhanced ACE and AngII receptor type 1 (AT1R) signaling through LPS [30]. In our recent study, we could demonstrate increased AngII levels in the serum of a mouse model of active colitis [31].

The role of AngII in atrial electrophysiological and structural remodeling is well-established [32,33]. AngII signaling through the AT1R promotes ROS production, changes in cellular Ca^2+^ handling properties, and the cellular AP [34]. However, whether a change in AngII signaling contributes to the observed alterations in atrial electrophysiology during active colitis remains to be determined.

In this study, using an animal model of dextran sulfate sodium (DSS)-induced active colitis, we aim to determine the mechanism of colitis-induced atrial electrophysiological remodeling and to test the hypothesis that a colitis-induced increase in AngII/AT1R signaling alters atrial ECG parameters by attenuating atrial conductance and prolonging the APD as a consequence of reduced I_Na_ and I_to_ availability, respectively.

## 2. Materials and Methods

### 2.1. Animals

**Figure 1:**
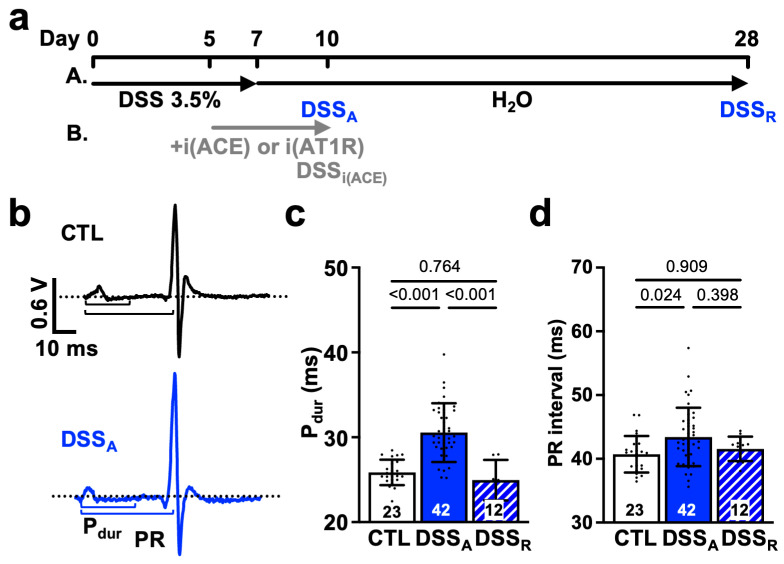
Active colitis attenuates atrial and AV nodal conductance: (**a**) Schematic representation of the treatment protocol for DSS-induced colitis (A). Experimental days for active colitis (DSS_A_) and remission (DSS_R_) are marked. Treatment with ACE inhibitor (B) Perindopril (i(ACE): ip: 3 mg/kg/day) or AT1R blocker Losartan (i(AT1R): ip: 30 mg/kg/day) is indicated in grey. (**b**) Representative ECG recordings obtained in anesthetized control (CTL) and DSS_A_ mice. Dotted black and purple lines show isoelectric lines; P-wave duration (P_dur_), and PR interval are illustrated by solid lines. Quantification of (**c**) P_dur_ and (**d**) PR interval from ECGs recorded in anesthetized CTL (animals: *n* = 23), DSS_A_ (*n* = 42), and DSS_R_ (*n* = 12) mice. Data are presented as mean ± SD, and significance was determined by one-way ANOVA followed by Tukey’s multiple comparison test (**c**,**d**).

Three- to six-month-old male mice (C57BL/6; The Jackson Laboratory, Bar Harbor, ME USA) were used for the study. Active colitis was induced by treating mice with DSS (MW: 36–50 kDa, MP Biomedicals, Irvine, CA, USA)-supplemented drinking water (3.5% weight/volume, 7 days; Figure 1a) as previously described [31]. For this purpose, CTL mice and DSS-treated mice were both supplied with water bottles one day before DSS supplementation. DSS-induced damage occurs predominantly in the distal part of the colon, reflecting the disease phenotype of ulcerative colitis [35]. Development of colitis was quantified in vivo as previously described using the animal’s body weight, stool consistency, and rectal bleeding to determine the disease activity index (DAI) [31,36]. Peak DAI at day 3 after DSS treatment was defined as active colitis (DSS_A_), whereas the return of DAI to the baseline 21 days after removal of DSS was defined as remission (DSS_R_) [31,37]. To evaluate the contribution of AngII signaling to the colitis-induced changes in cardiac electrophysiology, animals were treated with the ACE inhibitor Perindopril (intraperitoneal, ip: 3 mg/kg/day; Sellek Chemicals, Huston, TX, USA) [31,38] or the AT1R blocker Losartan (ip: 30 mg/kg/day; Sellek Chemicals, Huston, TX, USA) starting on day 5 of DSS treatment until sacrifice (DSS_i(ACE),_ DSS_i(AT1R)_, Figure 1a). CTL animals were treated with the drugs for the equivalent amount of time. All animal procedures were performed with the approval of the IACUC of Rush University (protocol code #22-034 approved 13 July 2022) and in accordance with the National Institute of Health’s Guide for the Care and Use of Laboratory Animals.

### 2.2. Electrocardiogram Recordings

In isoflurane-anesthetized mice (induction: 4%; maintenance: 2%; O_2_: 0.8–1.0 L/min), ECG recordings were performed using a Mouse Surgical Monitor (Indus Instruments, Webster, TX, USA) [39]. Data were digitized (4 kHz; PowerLab 8/35, AD instruments, Colorado Springs, CO, USA) and analyzed with LabChart 8 (AD instruments, Colorado Springs, CO, USA). ECGs were recorded continuously after a 10-min adaptation period. All ECG recordings were performed between 8:00–10:00 AM to minimize circadian variation in heart rate [31,39].

### 2.3. Isolated Langendorff Perfused Hearts

Mouse hearts were isolated, then a perfusion cannula was inserted into the aorta and connected to a Langendorff perfusion system (Harvard Apparatus, Holliston, MA, USA). The heart was continuously perfused with Krebs-Hänseleit solution containing (in mmol/L): 119 NaCl, 4 KCl, 1.2 KH_2_PO_4_, 25 NaHCO_3_, 10 Glucose, 2 Na Pyruvate, 2 MgSO_4_, 1.8 CaCl_2_; at 37 °C, 95% O_2_–5% CO_2_, pH of 7.4) [34]. Atrial electrograms were recorded using multielectrode arrays (FlexMEA36; Multichannel Systems, Reutlingen, Germany) placed on the left atrial epicardial surface. The array that consists of 36 gold electrodes with a diameter of 50 mm and an interelectrode distance of 300 mm was placed on the atrial epicardial surface. Atrial electrograms were recorded continuously during the adaptation period (15 min) and the experimental protocol. Electrograms were analyzed for their field potential duration (FP_dur_), FP dispersion (FP_disp_), and conduction velocity using Cardio2D+ (V 2.16.5, Multichannel Systems, Reutlingen, Germany) as previously described [31,39].

### 2.4. Current and Voltage Clamp Recordings

Patch clamp recordings (MultiClamp 700A, Axon Instruments, Molecular Devices, San Jose, CA, USA) on isolated atrial myocytes were performed at room temperature, in the whole cell, current- (APs) and voltage-clamp (Na- and K- currents, I_Na_, I_K_) configuration. For AP and I_K_ recordings, the extracellular solution contained (in mmol/L) 140 NaCl, 5 KCl, 1 MgCl_2_, 1 CaCl_2_, 10 D-Glucose, 10 HEPES, and pH = 7.4 (NaOH), and intracellular solutions contained (in mmol/L) 140 KCl, 5 NaCl, 4 MgATP, 5 EGTA, 10 HEPES, pH = 7.2 (KOH). To record I_Na_, the extracellular solution contained (in mmo/L) 130 NaCl, 10 CsCl, 1 MgCl_2_, 1 CaCl_2_, 10 D-Glucose, 10 HEPES, 0.005 Nifedipine, and pH = 7.4 (NaOH), and the intracellular contained (in mmo/L): 130 CsCl, 10 NaCl, 5 MgATP, 5 EGTA, 10 HEPES, and pH = 7.2 (CsOH). I_Na_ was further normalized to the maximum peak current (*I*_max_) and fit to a two-state Boltzmann distribution: *I*/*I*_max_ = (1 + exp[*ze*(*V* − *V*_1/2_)/*kT*])^−1^, where *V*_1/2_ is the potential of half-maximal activation (*V*_1/2a_) and inactivation (*V*_1/2i_), *k* is the Boltzmann constant, *z* is the apparent gating charge, *T* the absolute temperature, and *kT*/*e* = 25 mV at 22 °C.

For atrial myocyte AP recordings, a holding current was applied to maintain V_m_ at −85 mV, and APs were induced by current injection at a frequency of 1 Hz. Holding currents did not vary significantly between groups (CTL: −91.65 ± 47.86 pA; DSS_A_: −77.90 ± 32.59 pA; DSS_R_: −97.73 ± 73.26 pA), excluding differences in diastolic current. To quantify I_Na_ and I_K_ activation, cells were held at a holding potential of −80 mV, and hyper- and de-polarizing pulses of 500 ms duration were applied in 5 mV increments from −140 mV to +100 mV (protocol ^①^). The I_Na_ voltage-dependent inactivation was determined at the end of the test pulse by clamping the cell to −20 mV (50 ms), before return to the holding potential. Data were digitized (Digidata1322A at 10 kHz) and analyzed with Clampex 8.2 and Clampfit 11.2, respectively (Molecular Devices, San Jose, CA, USA). All whole-cell currents were normalized to cell capacitance (pA/pF) and are presented in dependence on membrane voltage (V_m_).

### 2.5. Isolation of Mouse Atrial Myocytes

Atrial myocytes were isolated by Langendorff perfusion and enzymatic digestion as previously described [40,41]. In short, the atria were dissected, cut into strips and incubated in digestion buffer (mg/L); 0.1 Liberase TM (Roche, Indianapois, IN, USA), 0.14 trypsin (Thermo Fisher Scientific, Waltham, MA, USA), and 1 Protease type XIV (Sigma-Aldrich Inc., St. Louis, MO, USA) for 20 min at 37 °C. Digestion was stopped by addition of bovine calf serum (Hyclone, Thermo Fisher Scientific, Waltham, MA, USA) before Ca^2+^ in the solution was reintroduced in a stepwise manner.

### 2.6. Protein Quantification by Western Blot

Protein quantification and immunoblotting were performed as previously described [34,39]. In short, freshly isolated atrial tissue was washed and rapidly frozen in liquid nitrogen. Tissue was lysed in hot 1-X- Laemmli sample buffer and protein quantified using a BCA protein assay kit (Thermo Fisher Scientific, Waltham, MA, USA). SDS-PAGE was performed on a 4–20% Tris-Glycine gel with 30–40 μg of protein loaded. After protein transfer, membranes were stained with Ponceau S for total protein quantification and incubated with primary antibodies overnight (4 °C) against Cx43 (1:20,000, C619, Sigma- Aldrich Inc. St. Louis, MO, USA) and SCN5A (1:10,000, ASC-005, Alamone). Species-specific horseradish peroxidase-conjugated secondary antibodies were used, and visualization was accomplished by Western Pierce^TM^ enhanced chemiluminescence (Thermo Fisher Scientific, Waltham, MA, USA). The Syngene PXi 6 imager in combination with the GeneSYS software (Syngene) was used for image acquisition. Changes in protein level were quantified with ImageJ version 1.54 (U.S. National Institutes of Health, Bethesda, MD, USA).

### 2.7. Chemicals

All reagents were purchased from Sigma Aldrich, except for LiberaseTM (Roche, Indianapois, IN, USA), Perindropil L-arginine (Sellek Chemicals, Huston, TX, USA), dextran sulfate sodium salt (MP Biomedicals, Irvine, CA, USA), trypsin (Thermo Fisher Scientific, Waltham, MA, USA), and bovine calf serum (Hyclone, Thermo Fisher Scientific, Waltham, MA, USA).

### 2.8. Statistic

A Shapiro-Wilk test was performed to assess the normal distribution of the data. Comparisons are made using a Student’s *t*-test (2 data groups) or one-way ANOVA (>3 data groups) with Tukey’s multiple comparison test. When the Shapiro–Wilk test revealed a non-parametric distribution, the one-way ANOVA on ranks, or Mann–Whitney U test were used as indicated. Data are presented when possible as scatter plots, as mean ± standard deviation (SD), or ±standard error mean (SEM) in case of the current voltage relationships. The number of experiments is provided as number of mice/hearts for ECG, Langendorff, and MEA recordings, or as the number of cells obtained from the respective number of isolations (cells/mice). The level of significance was set at *p* < 0.05.

## 3. Results

### 3.1. Change in Atrial Electrophysiological Properties During Active Colitis

**Figure 2:**
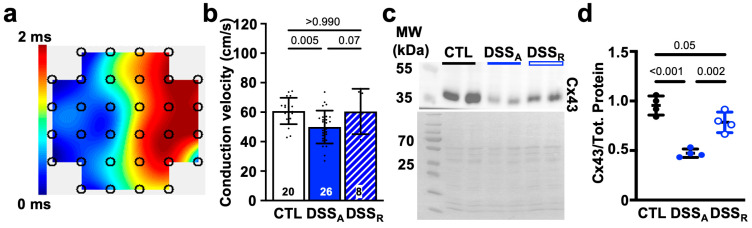
Active colitis attenuates atrial conduction velocity: (**a**) Representative contour plot from a left atrial MEA recording. (**b**) Conduction velocity quantified from atrial CTL (white; hearts: *n* = 20), DSS_A_ (blue; *n* = 26), and DSS_R_ (hatched; *n* = 8) MEA recordings. (**c**) Representative western blot of Cx43 (**top**) and Ponceau S staining (**bottom**) with molecular weight (MW) markers labeled on the left (four different left atrial tissue samples, two technical controls). (**d**) Quantification of western blot results (**c**) normalized to total protein. Data are presented as mean ± SD, significance was determined by one-way ANOVA followed by Tukey’s multiple comparison test.

Clinical studies show that patients with active colitis exhibit an increased propensity for AF [7,8,9]. To determine the mechanism of colitis-induced remodeling of atrial electrophysiological properties, we used a mouse model of DSS-induced active colitis and compared atrial ECG parameters from control (CTL) mice with those from mice with active colitis (DSS_A_) and in remission (DSS_R_; Figure 1a(A)). Despite comparable heart rates under basal conditions [31], DSS_A_ mice exhibited a prolonged P_dur_ and PR interval (Figure 1b–d). These changes in atrial electrophysiological properties were reversible upon remission. In vivo, the heart is under the persistent control of the autonomic nervous system. To determine if the changes in atrial electrophysiology are intrinsic to the heart (independent of the autonomic control), hearts were isolated, perfused in the Langendorff configuration, and MEAs were used for the recording of left atrial electrograms [39,42]. MEA recordings revealed an attenuated left atrial conduction velocity in DSS_A_ atria that recovered upon remission (Figure 2a,b). The data support that active colitis induces reversible atrial electrophysiological remodeling and that clinical observations of atrial electrophysiological changes are recapitulated in the DSS mouse model of active colitis.

### 3.2. Colitis-Induced Changes of the Atrial Conduction Velocity

**Figure 3:**
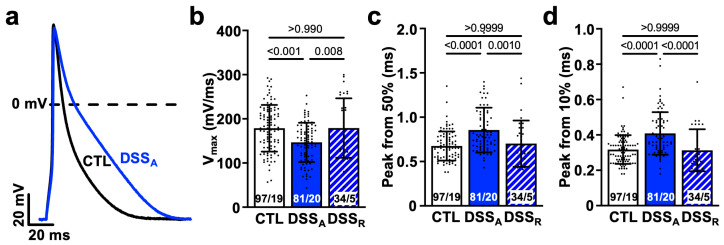
Active colitis attenuates atrial AP upstroke velocity: (**a**) Representative AP recordings (1 Hz) from CTL- and DSS_A_-isolated atrial myocytes. Quantification of AP (**b**) maximal upstroke velocity (V_max_) and rise time from (**c**) 50% and (**d**) 10% to peak amplitude. Numbers of cells and mice (cells/mice) are provided within columns. Data are presented as mean ± SD, significance was determined by one-way Anova on ranks and Dunn’s multiple comparison test.

Changes in atrial conduction velocity are often the consequence of an attenuated intercellular conductance established through the atrial gap junction proteins Cx40 and Cx43 [43,44], or an attenuated AP upstroke velocity caused by altered I_Na_ density or activation kinetic [45,46]. To determine if changes in atrial conductance depended on a change in intercellular coupling, we quantified left atrial Cx43 expression. A significant reduction in Cx43 protein was determined during active colitis that recovered upon remission (Figure 2c,d). To further evaluate changes in cellular excitability, we performed current clamp recordings in isolated atrial myocytes from CTL, DSS_A_, and DSS_R_ mice. AP recordings from atrial myocytes of DSS_A_ hearts displayed an attenuated maximal AP upstroke velocity (V_max_, Figure 3a,b), a delayed AP rise time (time from 50% or 10% to peak amplitude; Figure 3c,d), and an attenuated AP amplitude (APA) compared to myocytes from CTL and DSS_R_ mice (Supplemental Information (SI) Figure S1).

**Figure 4:**
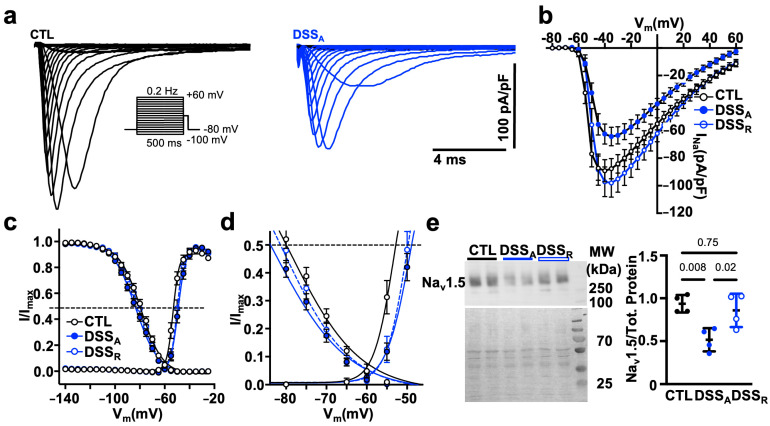
Active colitis alters atrial I_Na_ activation and inactivation kinetics: (**a**) Representative traces of I_Na_ density, recorded in atrial myocytes isolated from CTL (**left**) and DSS_A_ (**right**) mice, using the voltage protocol depicted. (**b**) Current-voltage relationship of I_Na_ peak current density from CTL (cells/mice: *n* = 37/6), DSS_A_ (*n* = 31/6), and DSS_R_ (*n* = 20/2) atrial myocytes. (**c**) Activation and inactivation kinetic of I_Na_/I_Na,max_ fitted with a two-state Boltzmann distribution. Dotted line indicates half-maximal activation (V_1/2a_) and inactivation (V_1/2b_), respectively. (**d**) Expansion of the plot shown in (**c**,**e**). Western blot of Na_v_1.5 (**top**) and Ponceau S staining (**bottom**, **left**) with molecular weight (MW) markers labeled on the left (four different left atrial tissue samples, two technical controls). Quantification (**right**) of Western blot results. Significance was determined by one-way ANOVA followed by Tukey’s multiple comparison test (**e**).

To determine if the attenuated AP upstroke velocity is a consequence of an altered I_Na_, current voltage relationships were recorded from isolated CTL, DSS_A_, and DSS_R_ atrial myocytes in the whole-cell voltage clamp configuration. To quantify the voltage dependent activation, I_Na_ was elicited from a holding potential of −80 mV, and the voltage dependent activation and inactivation was quantified. In DSS_A_ myocytes, the current density of I_Na_ (Figure 4a,b) was reduced and the voltages at half maximal activation (V_1/2a_) and inactivation (V_1/2i_) of I_Na_ were significantly shifted to more positive and negative voltages, respectively (Figure 4c,d, Table 1). The reduced I_Na_ density during active colitis was consistent with reduced protein levels of Na_v_1.5 quantified by western blotting (Figure 4e) that recovered during remission. The Na_v_1.5 protein ran slightly above the expected molecular weight, potentially reflecting the glycosylated form of the channel [47]. Interestingly, while I_Na_ density recovered to CTL levels upon remission, the activation and inactivation kinetic of the current remained attenuated compared to CTL. The experimental data suggest that active colitis attenuates atrial conduction velocity by reducing the availability of Na_v_1.5 and by attenuated Cx43 expression.

### 3.3. Colitis-Induced Changes of the Atrial APD

**Figure 5:**
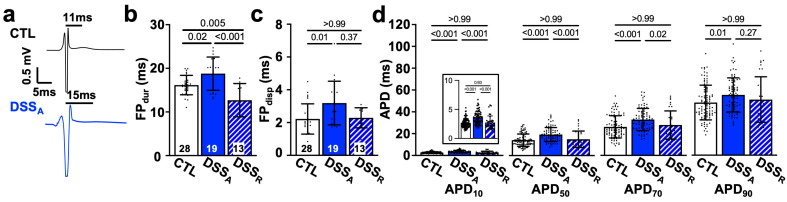
Active colitis alters atrial AP duration and dispersion: (**a**) Representative MEA recordings of atrial electrograms, black lines indicate duration of FP_dur._ (**b**) FP_dur_ as a measure of APD and (**c**) FP_disp_ quantified from MEA recordings of CTL (*n* = 28), DSS_A_ (*n* = 19), and DSS_R_ (*n* = 13) hearts. (**d**) AP duration (e.g., APD_10_) at 10%, 50%, 70%, and 90% amplitude recorded in CTL (cells/mice: *n* = 97/19), DSS_A_ (*n* = 81/20), and DSS_R_ (*n* = 34/5) atrial myocytes. Insert shows APD_10_ with expanded y-axis. Data are presented as mean ± SD, significance was determined by one-way Anova followed by Tukey’s multiple comparison test (**b**) or one-way Anova on ranks and Dunn’s multiple comparison test.

Alterations in atrial I_Na_ have been linked to an increased propensity for atrial arrhythmia because of a reduced atrial conduction velocity that facilitates the occurrence of reentry [23,24] or an increased I_Na,L_ that prolongs the APD and thereby increases the propensity for early afterdepolarization (EADs) and triggered activity [48]. To determine colitis-induced changes in the duration of cellular excitability, we quantified FP_dur_ from the atrial electrogram as a measure of the APD [31,42]. In mice with active colitis, atria exhibited a prolonged FP_dur_, and heterogeneity in atrial repolarization, quantified as FP_disp_, was increased (Figure 5a–c). The atrial data were supported by current clamp AP recordings on the cellular level. APDs were quantified at 10%, 50%, 70%, and 90% of repolarization (e.g., APD_10_; Figure 5d) and exhibited a significant prolongation during active colitis. The colitis-induced APD prolongation was reversible upon remission. However, the APD prolongation did not coincide with an increase in I_Na,L_ density or a change in the ratio between peak and steady-state I_Na_ (Figure S2a,b).

**Figure 6:**
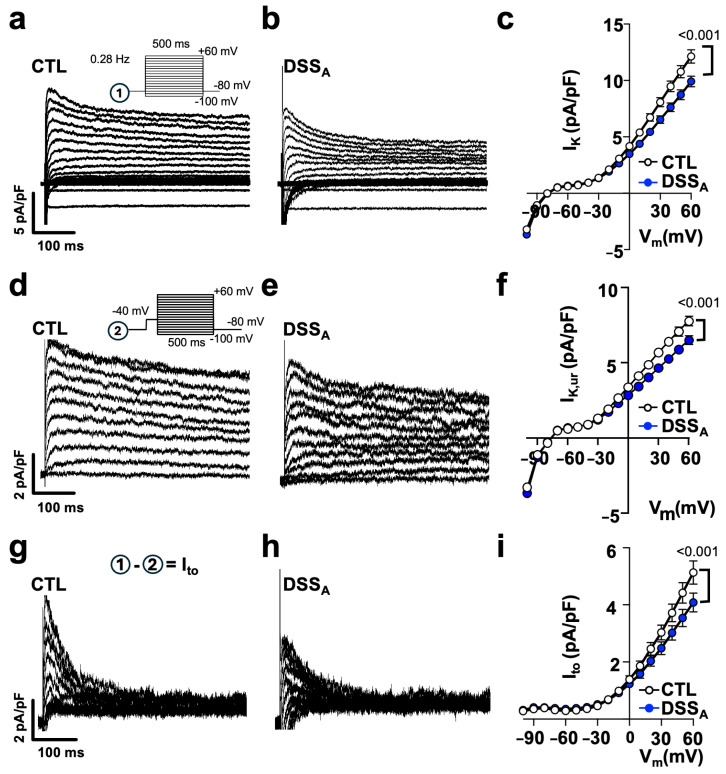
Active colitis reduces atrial I_K_ specifically I_to_: Representative traces of I_K_ density, recorded in atrial myocytes from (**a**) CTL and (**b**) DSS_A_ mice with voltage protocol ^①^ shown as inset. (**c**) I_K_ density vs. voltage (V_m_) relationship from CTL (cells/mice: *n* = 78/10) and DSS_A_ (*n* = 66/10) myocytes. Representative traces show atrial I_K,ur_ current density from (**d**) CTL and (**e**) DSS_A_ myocytes obtained with voltage protocol ^②^ shown as inset. (**f**) Current density vs. voltage relationship of I_K,ur_ from CTL (*n* = 78/10) and DSS_A_ (*n* = 66/10) myocytes. I_to_ obtained from (**g**) CTL and (**h**) DSS_A_ myocytes by subtraction of I^①^ − I^②^ = I_to_. (**i**) Current density vs. voltage relationship of I_to_ from CTL (*n* = 78/10) and DSS_A_ (*n* = 66/10) myocytes. Data are presented as mean ± SEM. Significance was determined by two-way ANOVA followed by Tukey’s multiple comparison test.

**Figure 7:**
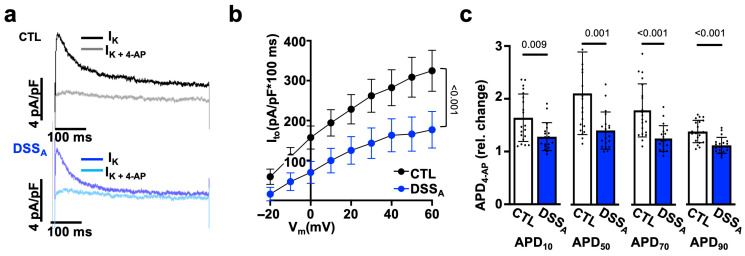
Active colitis reduces atrial I_K_: (**a**) Representative traces of atrial I_K_ current density recorded in CTL (**top**) and DSS_A_ (**bottom**) myocytes with protocol ^①^ at +40 mV, with and without 4-AP (100 µmol/L). (**b**) Quantification of the area under the curve (100 ms) of the 4-AP-sensitive I_K_(I_to_) in CTL (cells/mice: *n* = 23/5) and DSS_A_ (*n* = 20/6) atrial myocytes. (**c**) 4-AP-induced change in APD 10–90% in CTL (*n* = 27/8) and DSS_A_ (*n* = 25/7) myocytes. Data are presented as mean ± SEM (**b**) or mean ± SD (**c**), and significance was determined by two-way Anova (**b**) or Mann–Whitney test (**c**).

To determine differences in atrial myocyte repolarization, we quantified atrial K-currents using the voltage clamp technique. K-currents were elicited from a holding potential of −80 mV using the voltage protocol ^①^ depicted in Figure 6a. Active colitis induced a significant reduction in peak I_K_ in comparison to CTL atrial myocytes, suggesting a colitis-induced remodeling of fast voltage-activated K-channels. The early phase of AP repolarization is initiated by the voltage dependent activation of the slow and fast transient outward potassium currents (I_to,s_ and I_to,f_) as well as the ultra-rapid K-current (I_K,ur_). To isolate the contribution of I_to,f_ and I_to,s_ from other K-channels, the voltage protocol ^①^ (Figure 6a) was repeated with a depolarizing pre-pulse (−40 mV, 100 ms) before the test pulses (protocol ^②^: Figure 6d) to promote the voltage-dependent inactivation of I_to_ and isolate I_K,ur_ (Figure 6d,e). I_K,ur_ (Figure 6f), but also I_to_, quantified as the differential current (I_to_ = I^①^ − I^②^; Figure 6g–i), were significantly attenuated during active colitis. In an alternative approach, we used 4-Aminopyridine (4-AP: 100 mmol/L), a pan-K-channel blocker [19] that predominantly blocks I_to_ (I_to,s_: K_v_1.4; I_to,f_: K_v_4.2/4.3 and I_K,ur_: K_v_1.5), in the atria. Superfusion of atrial myocytes with 4-AP eliminated the differences in I_K_ between the CTL and DSS_A_ myocytes (Figure S3), and the differential current density quantified as the area under the curve (0–100 ms) before and after superfusion with 4-AP (I_to_ = I^①^ − I_(_^①^_+4AP)_) revealed a significantly reduced I_to_ during active colitis (Figure 7b). To resolve the impact that I_to_ has on the atrial AP, APs were recorded from isolated myocytes before and during 4-AP superfusion. 4-AP shortened the APD in CTL and DSS_A_ atrial myocytes; however, the degree of change was significantly larger in CTL myocytes (Figure 7c), consistent with the observed reduction of I_to_ density in DSS_A_.

To quantify the contribution of I_K,ur_ to the 4-AP sensitive current, I_K_ (protocol ^①^) was recorded before and during superfusion with Diphenyl phosphine oxide-1 (DPO-1: 1 mmol/L), an I_K,ur_ (K_v_1.5) specific blocker [49]. The DPO-1 sensitive current (I_K,DPO_ = I^①^ − I_(_^①^_+DPO)_) was significantly reduced during active colitis Figure S4a) consistent with the difference in current obtained with protocol ^②^ (Figure 6d–f). However, DPO-1 did not eliminate the difference in I_K_ between CTL and DSS_A_ myocytes (Figure S4b), and superfusion of DPO-1 during AP recordings induced comparable changes in APD of CTL and DSS_A_ myocytes (Figure S4c). Overall, the experimental data support a colitis-induced decrease in I_to_ (I_to,f_, I_to,s_, I_K,ur_) where the atrial APD prolongation is primarily a consequence of a reduction in I_to,f_.

### 3.4. Increased RAS Signaling Links Colitis to Atrial Remodeling

**Figure 8:**
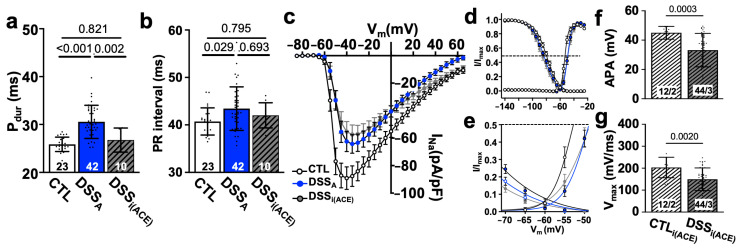
ACE inhibitor prevents electrophysiological remodeling. (**a**) P_dur_ and (**b**) PR interval quantified from ECGs recorded in CTL and DSS_A_ mice as well as mice treated with i(ACE) during the induction of colitis. (**c**) I_Na_ current density vs. voltage plot of CTL (cells/mice: *n* = 37/6), DSS_A_ (*n*= 31/6), and DSS_i(ACE)_ (*n* = 32/6) myocytes. (**d**) Activation and inactivation kinetic of I_Na_/I_Na,max_ fitted with two-state Boltzmann distributions of the data shown in (**c**). Dotted line indicates I_Na_ half maximal inactivation and activation, respectively. (**e**) Expansion of the plot shown in (**d**,**f**). AP amplitude (APA) and (**g**) V_max_ quantified from AP recordings (1 Hz) in isolated atrial myocytes. (**c**–**e**) Data are presented as mean ± SEM, the remaining as mean ± SD. Significance was determined by one-way ANOVA, followed by Tukey’s multiple comparison test.

**Figure 9:**
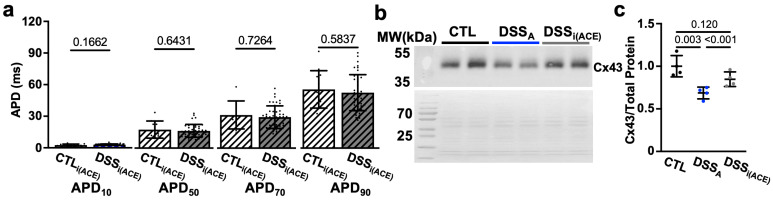
ACE inhibition during colitis (DSS_i(ACE)_) prevents APD prolongation: (**a**) APD at 10, 50, 70, and 90% of decay of CTL (cells/mice: *n* = 97/19), DSS_A_ (*n* = 81/20), and DSS_i(ACE)_ (*n* = 44/7) atrial myocytes. (**b**) Western blot (WB: **top**) of protein lysate from CTL, DSS_A_, and DSS_i(ACE)_, atria stained with antibody against Cx43 together with the corresponding Ponceau S staining. (**c**) Quantification of Western blot shown in (**b**) data normalized to total protein determined by Ponceau S stain (atrial samples: *n* = 4, repeated twice). Data are presented as mean ± SD. Significance was determined by one-way ANOVA (**a**,**b**), followed by Tukey’s multiple comparison test.

In patients, during active colitis, gut dysbiosis, bacterial translocation, inflammation, and altered autonomic control can enhance systemic AngII signaling [27,30], and our prior studies demonstrated increased serum levels of AngII in our mouse model of active colitis [31]. To determine if AngII contributes to the observed changes in atrial electrophysiology, we treated mice with the ACE inhibitor Perindopril (3 mg/kg/day, ip) during the DSS treatment period as previously described (Figure 1a(B)) [31]. At the end of treatment, ECGs were recorded and atrial myocytes isolated from ACE inhibitor (i(ACE)) treated mice (DSS_i(ACE)_). ECG analysis revealed that i(ACE) treatment during active colitis prevented a significant prolongation of P_dur_ (Figure 8a), and the PR interval remained comparable to CTL animals (Figure 8b). Interestingly, after i(ACE) treatment, I_Na_ remained attenuated (Figure 7c) and half maximal activation and inactivation remained significantly slower in DSS_i(ACE)_ compared to CTL myocytes (Figure 8d,e; Table 2), despite the ECG normalization and comparable expression levels of Na_v_1.5 in CTL- and iACE-treated animals (Figure S5). Consistent with the reduced I_Na_, the AP amplitude and upstroke velocity (APA, V_max_; Figure 8f,g) remained attenuated in DSS_i(ACE)_ myocytes, while APD shortening was prevented compared to CTL_i(ACE)_ myocytes (Figure 9a), especially during the early phase of repolarization. Comparison of Cx43 expression in CTL_i(ACE)_ and DSS_i(ACE)_ atria showed that Cx43 downregulation during active colitis was prevented by i(ACE) treatment (Figure 9b,c).

ACE inhibition prevents the conversion of AngI to AngII. To determine if the signaling observed is a consequence of enhanced AngII-induced AT1R activation, we repeated part of the experiments in CTL and DSS mice treated with the AT1R blocker Losartan-K (ip: 30 mg/kg/day). Comparable to iACE, block of AT1R during the induction of colitis (Figure 1a(B)) also prevented prolongation of the P_dur_ and PR interval (Figure S6a,b). Interestingly, in DSS_i(AT1R)_ myocytes, APA and V_max_ (Figure S6c,d), as well as the AP rise time (time from 50% and 10% to peak), were maintained, suggesting the prevention of I_Na_ remodeling (Figure S6e,f). Also, a DSS-induced prolongation of the APD was successfully suppressed by i(AT1R) (Figure S6g). Overall, the results support a role of increased AngII signaling in the deregulation of atrial electrophysiological properties during active colitis.

## 4. Discussion

In the present study, we demonstrate for the first-time on the cellular level an active colitis-induced atrial electrophysiological remodeling. We establish that P_dur_ and PR prolongation are the consequence of a reduced availability of Cx43 and the voltage dependent Na-channels (I_Na_, *SCN5A*), and that a prolongation of the APD is the result of a colitis-induced attenuation of I_to_. The observed atrial electrophysiological changes that are linked to an increased risk for atrial arrhythmia, were only partially reversible upon remission and the consequence of a colitis-induced increase in AngII signaling.

### 4.1. Atrial Remodeling in Patients with Active Colitis

Inflammatory bowel disease, including ulcerative colitis, is a common chronic inflammatory disorder of the gastrointestinal tract [1,2]. In the course of the disease, repeated episodes of acute inflammation become superimposed upon a chronic disease progression. Besides gastrointestinal symptoms, patients are reported to develop an increased risk for extraintestinal disorders [3]. Changes in cardiovascular function include supraventricular arrhythmia, AF, and an increased risk for stroke [7,8,9]. Interestingly, colitis patients present with new onset AF predominantly during the active phase of the disease [8,9]. To determine the mechanism of active colitis-induced changes in atrial electrophysiology that could increase the risk for atrial arrhythmia, we chose a mouse model of DSS-induced colitis [35,36]. Administration of DSS through drinking water causes injury to the intestinal epithelial lining [37]. It is well documented that the resulting transition of luminal bacteria and antigens into the mucosa results in an inflammatory response and the increase in inflammatory markers in the intestine (TNF-α, IL-1β, IFN-γ and cytokines) and serum (C-reactive protein) [50]. Colitis disease severity depends on the mouse strain, the concentration of DSS, as well as the duration and frequency of exposure [35,37]. In our study, the chosen DSS concentration of 3.5% induces the disease phenotype of active colitis [31], and the C57BL/6 strain was chosen because it develops a more severe and chronic disease phenotype more representative to human colitis. Our experimental results reveal a P-wave and PR interval prolongation that mirrors the atrial electrophysiological changes described in colitis patients [11,13,14] and highlights the usefulness of the model in studying the mechanism of colitis-induced atrial remodeling.

### 4.2. Active Colitis-Induced Prolongation of P_dur_ and PR Interval

The P-wave is a measure of the excitation spread across the atria, whereas P-wave dispersion reflects the homogeneity of inter-atrial conductance and/or propagation of the excitation across the atria [17,18]. Consistent with patient data, our results show a prolongation of P_dur_ and PR that coincided with a reduced atrial epicardial conduction velocity (Figure 2a,b) and increased dispersion of excitation (Figure 5c). The fact that the alterations in atrial excitation were reproduced in isolated hearts suggests that they are intrinsic to the atrial tissue and not a consequence of disease-dependent alterations in autonomic control or electrolyte imbalance [51]. On the tissue level, an attenuated cardiac conduction velocity can be the result of a decreased AP upstroke velocity, increased intercellular resistance, or structural remodeling such as tissue fibrosis [52]. Activation of atrial fibroblasts and fibrosis can be a consequence of an atrial inflammatory response [53], as it occurs in the DSS model of active colitis [35,50]. In our model, the reversibility of the slowed atrial conductance upon remission (Figure 2a) does not advocate for permanent structural fibrotic remodeling of the atria. The observed reversibility of the ECG changes stands in contrast to clinical data showing persistent ECG changes in patients in remission [6,11]. However, as described in the literature, these changes correlate with disease severity and duration and could suggest that in our model, longer or repeated episodes of active colitis could promote progressive and irreversible structural remodeling of the atria.

The AP upstroke velocity is driven by the activation of Na_v_1.5 (*SCN5A*) [54]. Altered Na_v_1.5 expression as well as gain and loss of function mutations have been linked to an increased propensity for AF [23,24]. Reduced current density as well as a shift of the channels voltage dependence of activation thereby impair atrial conduction and lead to a prolonged or a more heterogeneous excitation of the atria [21,55]. Our AP recordings in isolated atrial myocytes support an attenuated AP up-stroke velocity because of a reduction in Na_v_1.5 protein and a shift of the I_Na_ activation kinetic to more depolarizing voltages (Figure 3). While the conduction velocity, I_Na_ density, and protein levels recovered in remission, I_Na_ kinetic remained attenuated, suggesting that the change in kinetic alone was insufficient to cause the slowed atrial conduction velocity. The latter is not surprising given that changes in intercellular coupling were shown to have a more pronounced impact on cardiac conduction velocity than alterations in I_Na_ [20]. Attenuated atrial gap junction coupling due to a reduction in channel expression, a change in the channel’s sub-cellular distribution, or mutation-dependent changes in the channel’s conductance were shown to attenuate atrial conductance. The colitis-induced, reversible reduction in the protein level of Cx43 is therefore more likely the determining factor for the reduced atrial conduction velocity in DSS_A_ atria. At this point, we do not know if the recovery of I_Na_ activation kinetic during remission is slowed or if the changes remain permanent. In the latter case, repeated disease episodes could progressively and persistently impact atrial conduction and dispersion of excitation, even in remission.

### 4.3. Colitis-Induced Changes in APD

Changes in the atrial APD are linked to an increased risk for atrial arrhythmia [52,56]. During rapid atrial pacing, or in the context of AF, a shortened APD attenuates the atrial refractory period and facilitates the occurrence of reentry or delayed after depolarizations (DADs) [52]. On the other hand, a prolongation of the atrial APD can increase the risk for arrhythmic events by facilitating the incidence of EADs. A prolongation of the atrial APD is the result of increased depolarizing currents, such as raised levels of I_Na,L_, the L-type Ca-current, or enhanced sodium calcium exchanger (I_NCX_) activity [48]. Alternatively, a prolongation of the APD can be caused by a reduction in repolarizing K-currents. Atrial K-currents are composed of a large group of voltage-regulated channels with different activation kinetics [57,58]. I_to,f_ and I_to,s_ as well as I_K,ur_ (K_v_1.5) are rapidly activated during the upstroke of the AP and regulate the APs amplitude as well as duration. The downregulation of I_to_ [59,60] and I_K,ur_ [57,61] have been described in patients as well as animal models of AF, and mutations have been linked to heritable cardiac arrhythmia. The colitis-induced prolongation of the early phase of the atrial APD (APD_10_, APD_50_) suggests an attenuation of I_to,f_, I_to,s_, and I_Kur_. This was supported by our experimental data, that show significantly reduced 4-AP and DPO-1 sensitive currents in DSS_A_ myocytes (Figure 7b, Figure S4a) and the elimination of differences in I_K_ between CTL and DSS_A_ after the pharmacological inhibition of I_to_ (Figure S3). The failure of DPO-1 to eliminate differences in APD between CTL and DSS_A_ myocytes insinuates that the atrial-specific I_K,ur_ (K_v_1.5) has a more limited impact on APD prolongation during active colitis.

The fast and slow component of atrial I_to_ refers to the current’s inactivation and recovery from inactivation, respectively. I_to,f_ has been linked to the K_v_4 family K_v_4.2 and K_v_4.3 (*KCND2/3*), whereas I_to,s_ has been linked to K_v_1.4 (*KCNA4*) [62]. Interestingly, K_v_1.4 is expressed in a species-dependent manner and shows a spatially defined expression pattern [62]. In rabbits and humans, the K_v_1.4 protein was determined in the atrial muscle, whereas in mice, deletion of K_v_1.4 had no impact on the atrial electrophysiology but increased the occurrence of AV block and altered the ventricular APD [63]. In mouse atria however, the expression of a dominant negative form of K_v_4.2 eliminated I_to_, suggesting that K_v_4 channels are a major contributor to early AP repolarization [64]. At this point, we therefore suggest that the colitis-induced APD prolongation is predominantly the result of a reduction of I_to,f_ (K_v_4.2,4.3)-dependent currents.

### 4.4. The Gut-Heart Axis

Gut dysbiosis, colonic barrier disruption with bacterial translocation, increased sympathetic tone and increased levels of systemic inflammation in symptomatic patients with active ulcerative colitis [3,65]. A link between gut health and cardiovascular function has been previously demonstrated. Therefore, the progression of heart failure, atherosclerosis, cardio-metabolic syndrome, hypertension, and endocarditis have been linked to changes in gut microbiota, transfer of bacteria into the circulation, as well as changes in bacterial metabolites [66]. More recently, increasing evidence has emerged of a close link between AF and gut dysbiosis [26]. The mechanistic link, however, remains complex and underexplored. Potential mechanisms are increased levels of LPS and TMAO metabolic products of the gut microbiome, as well as decreased levels of SCFAs, such as butyrate [26]. In all cases, their dysregulation was linked to increased inflammatory signaling and an increased propensity of cardiac fibrosis [26,67]. One commonality of all signaling mechanisms is their impact on RAS signaling [68]. Increased levels of inflammatory cytokines increase Agt expression [29], while LPS enhances ACE and AT1 signaling. SCFAs on the other hand, attenuate renin secretion [30]. Colitis-induced inflammation, an increase in LPS, the sympathetic tone, and a decrease in SCFA would all result in increased levels of Agt and AngII [69,70], which have been shown to correlate with disease activity [71]. AngII itself, which we have shown to be increased in the serum of our mouse model of active colitis [31], can further intestinal and systemic inflammation by stimulating the expression of inflammatory cytokines. The latter is consistent with the observation that in AT1R-deficient mice and during i(ACE) treatment [72], colitis-induced intestinal inflammation is attenuated [73].

In the heart, acute and chronic exposure to AngII promotes electrophysiological remodeling, hypertrophic growth, ROS signaling, and fibrotic remodeling [74]. Increased AngII levels were determined in the atria of patients with AF, and in mouse models, chronic AngII exposure or overexpression of AT1R increased P_dur_, atrial effective refractory period, and the propensity for AF [19,75]. The AngII-induced attenuation of the atrial conduction velocity has been linked to reduced I_Na_, Cx43, and Cx40, whereas the AP prolongation coincided with an attenuation of I_to_ and I_K,ur_ [19,75,76]. In our model of active colitis, we identified an atrial electrophysiological phenotype that exhibits a prolonged P_dur_ and attenuated conduction velocity based on a reduced Cx43 expression and I_Na_ availability, whereas the observed APD prolongation is a consequence of attenuated repolarizing I_to,f_. The remodeling of the conduction velocity and APD could be prevented by treatment of mice with i(ACE) (Figure 8 and Figure 9) or i(AT1R) (Figure S6) during the induction of colitis. While the effect of i(ACE) only supports an increase in RAS signaling, the protective effect of i(AT1R) narrows the signaling down to an AngII/AT1R-induced signaling cascade. At this point, we do not distinguish if the colitis-induced increase in AngII signaling is caused by changes in the autonomic tone, inflammation, or alterations in gut microbiome and its metabolites. We also cannot rule out an attenuation of signaling pathways that would counteract signaling downstream of AT1R; however, the effectiveness of i(ACE) and i(AT1R) underlines the observed contribution of this receptor mediated pathway in the atrial remodeling.

### 4.5. Limitations

DSS, as well as other colitis models, are indispensable tools to study IBD, but no model reflects the complexity of the human disease [77]. In contrast to IBD in humans, inflammation induced by DSS is more severe and occurs more rapidly. In addition, in mice, T- and B- cells are not required for the development of the disease and intestinal bacteria differ from those in humans. We chose the model of DSS-induced colitis because it can mimic acute, chronic, and relapsing phases of the disease, and the induced dysplasia resembles the clinical phenotype of human ulcerative colitis. Also, cytokines (IL-6, -16, -22) and chemokines (CCL2, CCL3, CXCL1) related to human IBD are upregulated [50]. In future studies, we will further verify our findings in other colitis models (e.g., spontaneous colitis, inducible colitis, genetically modified, and adoptive transfer models).

The model of acute colitis was chosen because IBD patients with acute colitis exhibit a more than two-fold increase in their risk for AF. Disease severity in patients, however, will most likely be impacted by the chronic progression of the disease. The fact that electrophysiological remodeling occurs during the first appearance of acute colitis underlines the significance to better understand the gut-heart axis. In the future, atrial remodeling in models of chronic disease progression will elucidate the long-term impact of the disease on the heart.

A Mendelian randomization study has argued against a causative link between colitis and AF [78]. However, limitations of the studies were that IBD disease activity, severity, and duration were not included in the analysis, and the occurrence of AF was not evaluated by long-term monitoring and therefore likely underestimated. Our data support atrial electrophysiological remodeling as a consequence of colitis; however, further clinical and animal studies are warranted to better link IBD disease activity to changes in atrial electrophysiology and the propensity for AF.

## 5. Conclusions

In the presented study, we demonstrate for the first time that active colitis induces atrial electrophysiological remodeling, which creates an atrial substrate described to have an increased propensity for arrhythmia. The colitis-induced increase in AngII signaling attenuated the conduction velocity and increased APD through the remodeling of I_Na_, intercellular coupling, and I_to,f._ The results are consistent with clinical reports showing an increased vulnerability of colitis patients to atrial arrhythmia, and suggest that in especially hypertensive colitis patients, ACE inhibitors should be the treatment of choice, and increased RAS signaling could serve as a marker for colitis patients at risk for atrial arrhythmia.

## Figures and Tables

**Table 1:** Changes in Boltzmann parameters for I_Na_ activation and inactivation kinetic during colitis.

		CTL	DSS_A_	DSS_R_	*p* Values		
Activation	n	37/6	31/6	20/2	CTL vs. DSS_A_	CTL vs. DSS_R_	DSS_A_ vs. DSS_R_
	V_1/2a_	−53.3 ± 0.2	−49.2 ± 0.2	−50.1 ± 0.2	<0.001	<0.001	0.025
	slope	2.5 ± 0.2	3.0 ± 0.2	2.6 ± 0.2	0.096	0.932	0.327
Inactivation	n	37/6	31/6	20/2			
	V_1/2i_	−79.2 ± 0.4	−83.3 ± 0.3	−80.7 ± 0.5	<0.001	0.024	<0.001
	slope	−8.7 ± 0.3	−9.1 ± 0.3	−8.2 ± 0.4	0.535	0.634	0.179

**Table 2:** ACE inhibitor prevents changes in Boltzmann parameters for I_Na_ activation and inactivation kinetic during colitis.

		CTL	DSS_A_	DSS_i(ACE)_	*p* Values		
Activation	n	37/6	31/6	32/6	CTL vs. DSS_A_	CTL vs. DSS_i(ACE)_	DSS_A_ vs. DSS_i(ACE)_
	V_1/2a_	−53.3 ± 0.2	−49.2 ± 0.2	−49.0 ± 0.3	<0.001	<0.001	0.827
	slope	2.5 ± 0.2	3.0 ± 0.2	3.8 ± 0.2	0.162	<0.001	0.006
Inactivation	n	37/6	31/6	32/6			
	V_1/2i_	−79.2 ± 0.4	−83.3 ± 0.3	−85.8 ± 0.4	<0.001	<0.001	<0.001
	slope	−8.7 ± 0.3	−9.1 ± 0.3	−8.9 ± 0.4	0.727	0.997	0.785

## Data Availability

The data that support the findings of this study are available from the corresponding author upon reasonable request.

## Supplementary Materials

The following supporting information can be downloaded at: https://www.mdpi.com/article/10.3390/biom15070982/s1, Figure S1: Colitis attenuates AP amplitude; Figure S2: I_Na,L_ does not contribute to colitis-induced AP prolongation; Figure S3: 4-AP relinquishes the difference in I_K_ during active colitis; Figure S4: DPO does not relinquishes the difference in I_K_ during active colitis; Figure S5: AT1R block does not prevent Na_v_1.5 downregulation; Figure S6: AT1R block during colitis prevents atrial electrophysiological remodeling.

## Abbreviations

The following abbreviations are used in this manuscript:

ACE	Angiotensin-converting enzyme
AF	Atrial fibrillation
Agt	Angiotensinogen
AngII	Angiotensin II
AP	Action potential
APD	Action potential duration
AT1R	Angiotensin II receptor type 1
Cx	Connexin
DAI	Disease activity index
DSS	Dextran sulfate sodium
ECG	Electrocardiogram
IBD	Inflammatory bowel disease
IL	Interleukin
LPS	Lipopolysaccharide
LTCC	L-type Ca channel
Pdur	P wave duration
Pdisp	P wave dispersion
RAS	Renin-angiotensin system
SCFA	Short chain fatty acids
TMAO	Trimethylamine N-oxide
UC	Ulcerative colitis
