# Supplementary material for: Active Colitis-Induced Atrial Electrophysiological Remodeling
Source: Biomolecules. 2025 Jul 10;15(7):982. doi: 10.3390/biom15070982 (PMC12292842; doi:10.3390/biom15070982)
Supplement: Supplementary file 1 [file biomolecules-15-00982-s001.zip › biomolecules-3698844-supplementary.pdf]

Supplementary Materials

Figure S1

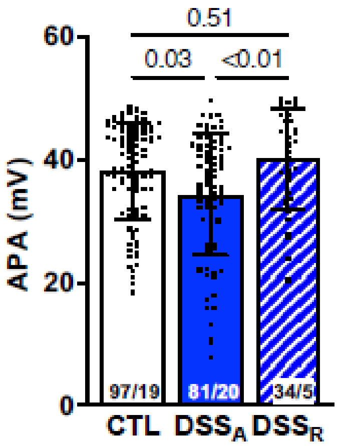

Figure S1 Colitis attenuates AP amplitude

APA quantified from AP recordings (1 Hz) in CTL, DSS_A, and DSS_R myocytes. Number of cells/mice are provided inside the column. Data are presented as mean ± SD and significance was determined by one-way ANOVA followed by Tukey's multiple comparison test.

Figure S2

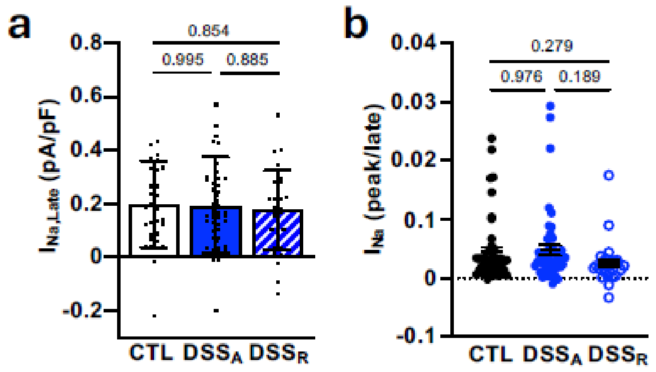

Figure S2 $I_{Na,L}$ does not contribute to colitis-induced AP prolongation

a. In atrial myocytes of CTL (*n* = 46/6), DSS_A (*n* =53/8) and DSS_R (*n* = 31/4) mice no changes in $I_{Na,L}$ density or **b.** in the ratio between peak and steady state current were determined. Data are presented as mean ± SD and significance was determined by one-way ANOVA followed by Tukey's multiple comparison test.

Figure S3

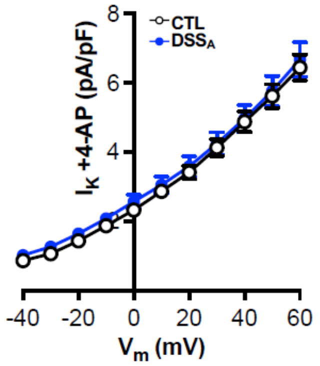

Figure S3 4-AP relinquishes the difference in IK during active colitis
IK, recorded in the presence of 4-AP (100 mmol/L) in CTL (cells/mice: *n* = 23/5) and DSS_A (*n* = 21/6) isolated atrial myocytes. Data are presented as mean ± SEM.

Figure S4

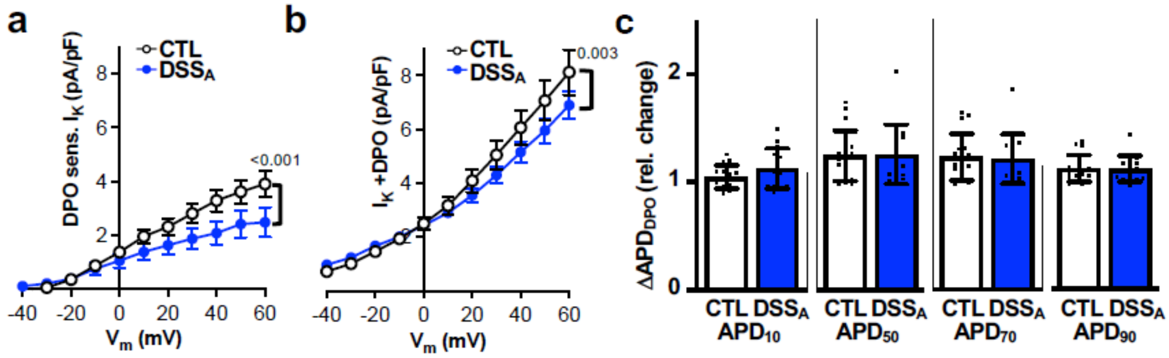

Figure S4 DPO does not relinquishes the difference in I_K during active colitis
a. IK, recorded in the presence of 4-AP (100 mmol/L) or **b.** DPO-1 (1 mmol/L) in CTL (cells/mice: *n* = 23/5; 21/4) and DSS_A (*n* = 21/6; 17/4) isolated atrial myocytes. **c.** DPO-1 induced change in APD at 10% to 90% inactivation in CTL (*n* = 19/4) and DSS_A (*n* = 14/4) myocytes. Data are presented as mean ± SEM (**a**,**b**) or ± SD (**c**). Significance was determined by two-way ANOVA (**a**,**b**) or Mann-Whitney test (**c**)

Figure S5

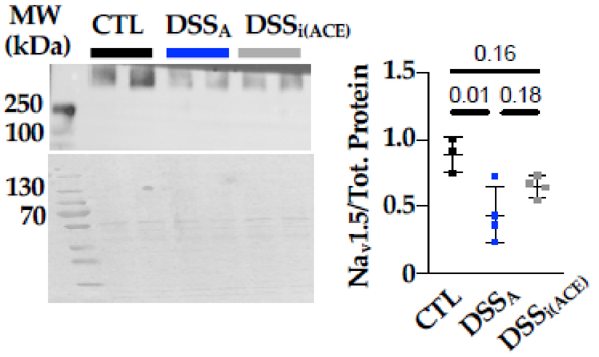

Fig. S5 AT1R block does not prevent $Na_v1.5$ downregulation
a. Western blot of $Na_v1.5$ (top) and Ponceau S staining (bottom) with molecular weight (MW) markers labeled on the left (4 different left atrial tissue samples, 2 technical controls). Data are presented as mean ± SD and significance was determined by one-way ANOVA followed by Tukey' s multiple comparison test.

Figure S6

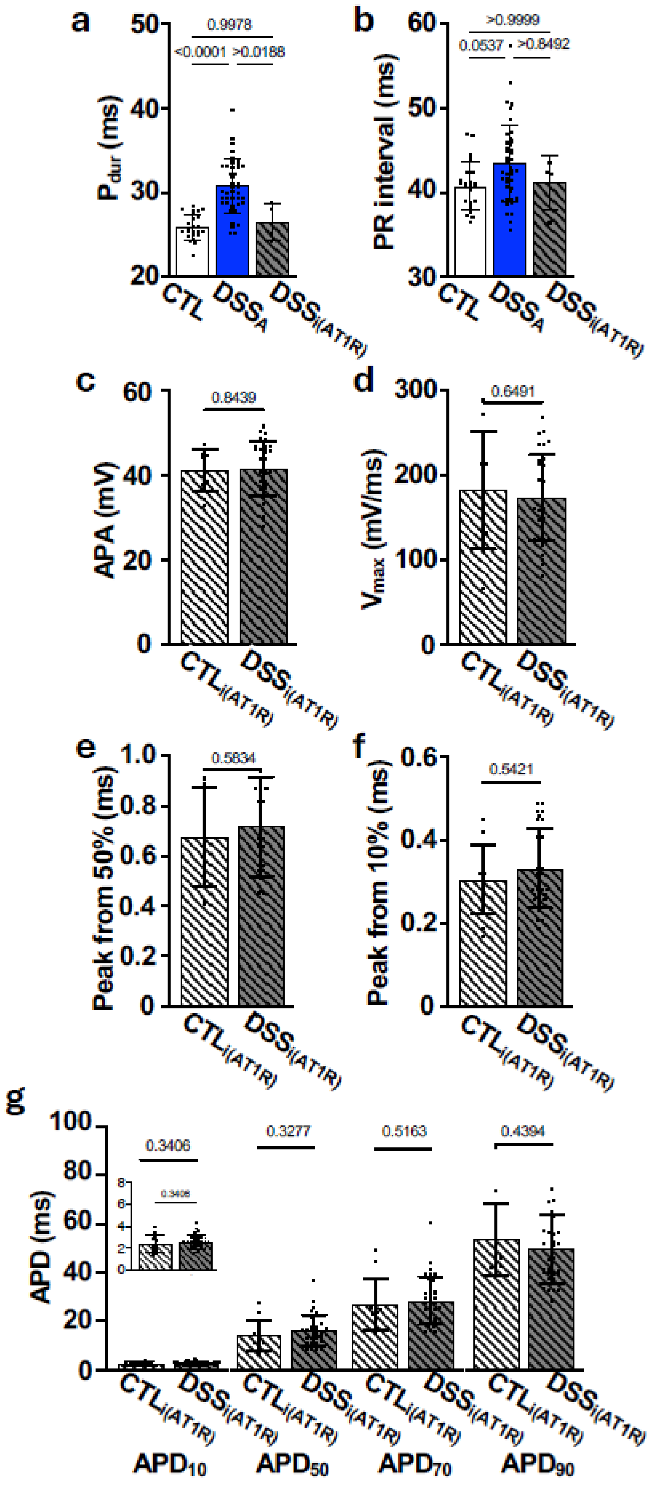

Fig. S6 AT1R block during colitis prevents atrial electrophysiological remodeling

a. P_{dur}′ and **b.** PR interval from ECGs recorded in anesthetized CTL (*n*=23), DSS_A (*n*=42), and $DSS_{i(AT1R)}$ (*n*=4) mice. AP quantification of **c.** APA, **d.** V_{max}, time to peak from **e.** 50% and **f.** 10% amplitude as well as **g.** AP duration (APD) at 10% (insert shows data on extend y-axis), 50%, 70% and 90% amplitude recorded in CTL (cell/mice: *n*= 97/19), DSSA (*n*= 81/20), and $DSSi_{(AT1R)}$ (*n*= 35/4) atrial myocytes. Data are presented as mean ± SD and significance was determined by unpaired Student's t- test.
